# Predicting grain yield using canopy hyperspectral reflectance in wheat breeding data

**DOI:** 10.1186/s13007-016-0154-2

**Published:** 2017-01-03

**Authors:** Osval A. Montesinos-López, Abelardo Montesinos-López, José Crossa, Gustavo de los Campos, Gregorio Alvarado, Mondal Suchismita, Jessica Rutkoski, Lorena González-Pérez, Juan Burgueño

**Affiliations:** 1 International Maize and Wheat Improvement Center (CIMMYT), Apdo. Postal 6-641, 06600 Mexico City, Mexico; 2 Facultad de Telemática, Universidad de Colima, 28040 Colima, Colima Mexico; 3 Departamento de Estadística, Centro de Investigación en Matemáticas (CIMAT), 36240 Guanajuato, Guanajuato Mexico; 4 Epidemiology and Biostatistics Department, Michigan State University, 909 Fee Road, East Lansing, MI 48824 USA; 5 International Rice Research Institute, Los Baños Research Center, Los Baños, Laguna Philippines

**Keywords:** Spectral data, Vegetation indexes, Prediction accuracy, Genome selection, Bayes B, Spline regression, Fourier regression, Wheat

## Abstract

**Background:**

Modern agriculture uses hyperspectral cameras to obtain hundreds of reflectance data measured at discrete narrow bands to cover the whole visible light spectrum and part of the infrared and ultraviolet light spectra, depending on the camera. This information is used to construct vegetation indices (VI) (e.g., green normalized difference vegetation index or GNDVI, simple ratio or SRa, etc.) which are used for the prediction of primary traits (e.g., biomass). However, these indices only use some bands and are cultivar-specific; therefore they lose considerable information and are not robust for all cultivars.

**Results:**

This study proposes models that use all available bands as predictors to increase prediction accuracy; we compared these approaches with eight conventional vegetation indexes (VIs) constructed using only some bands. The data set we used comes from CIMMYT’s global wheat program and comprises 1170 genotypes evaluated for grain yield (ton/ha) in five environments (Drought, Irrigated, EarlyHeat, Melgas and Reduced Irrigated); the reflectance data were measured in 250 discrete narrow bands ranging between 392 and 851 nm. The proposed models for the simultaneous analysis of all the bands were ordinal least square (OLS), Bayes B, principal components with Bayes B, functional B-spline, functional Fourier and functional partial least square. The results of these models were compared with the OLS performed using as predictors each of the eight VIs individually and combined.

**Conclusions:**

We found that using all bands simultaneously increased prediction accuracy more than using VI alone. The Splines and Fourier models had the best prediction accuracy for each of the nine time-points under study. Combining image data collected at different time-points led to a small increase in prediction accuracy relative to models that use data from a single time-point. Also, using bands with heritabilities larger than 0.5 only in Drought as predictor variables showed improvements in prediction accuracy.

## Background

Plant breeding programs routinely perform early field evaluations of large numbers of candidates for selection based not only on the main primary trait—grain yield measured in different environments—but also on several secondary traits related to yield, such as disease resistance. Methods that could help breeders measure grain yield based on other secondary traits in the early stages of plant growth could be of value to help reduce evaluation time and cost [25]. In recent years, the use of remote or proximal sensing, hyperspectral imaging, and laser scanners has helped develop low-cost, efficient high-throughput phenotyping platforms (HTPP) [1] which aim to collect data at low cost on many phenotypes of large numbers of breeding individuals at different stages of plant growth under different environmental conditions. This could drastically increase the number of traits that can be quantified on field-grown plants, selection intensity, prediction accuracy, and, therefore, the response to selection [25].

The basis of remote sensing and spectral science is the ability to measure electromagnetic energy at varying wavelengths that interact with different parts of the growing plant. The goal of spectral science is to measure a phenotype quantitatively through the interaction between light and plants, such as reflected, absorbed, transmitted and/or emitted photons. This is possible because each component of plant cells and tissues has wavelength-specific absorbance, reflectance and transmittance properties [16]. For example, a healthy plant interacts (absorbs, reflects, emits, transmits and fluoresces) with electromagnetic radiation in a different way than an infected plant [16].

In practical applications of HTPP to agriculture, the reflectance of electromagnetic energy at different wavelength bands is usually summarized in scores of spectral vegetative indices (VI) that are further used to predict plant physiological issues or agronomic traits. Spectral VI are a simple and convenient way of extracting information from remotely sensed data that facilitates the processing and analysis of large amounts of data acquired by modern cameras and satellite platforms [13, 18]. Significant advances have been achieved in understanding the nature and proper interpretation of spectral VI [18, 21] and theoretical frameworks have been proposed to support the development of indices optimized for particular applications. Popular VIs are the Normalized Difference Vegetation Index (NDVI), the Canopy Water Mass Index [30] and the Modified Normalized Difference at 705 nm wavelength (mND; e.g., [26]). Vegetative indices have been applied successfully in some crops [3–9, 31]. For example, the canopy temperature (CT) and NDVI indices have been applied to estimate yield, taking advantage of the correlation between yield and these two VIs [2, 15, 17, 22]. Also, it has been documented that the air-canopy temperature difference index can be used as a selection criterion in wheat (*Triticum aestivum* L.) breeding programs to estimate yield [24]. The NDVI has also been used successfully to estimate wheat yield before harvest at the regional and farm scale [15].

Although several spectral VIs are positively correlated with grain yield and other important agronomic and physiological traits, they do not consider all the spectral bands from the hyperspectral sensors [28, 29]. Nevertheless, cameras with high spectral resolution can produce data on hundreds of spectral bands that can be further used to capture a wide range of information. Also, despite some successful applications of spectral VIs, most of these indices tend to be species-specific and, therefore, are not robust when applied across different species that have different canopy architectures and leaf structures because they use only a fraction of the available information on the measured wavelengths.

The important idea is that for sensor data to be meaningful, algorithms need to be developed to interpret the data and extract the most useful information to be translated into important traits for plant breeding. Thus, the use of high-resolution images is important to develop prediction models for grain yield, yield components, and relevant physiological and agronomical traits. However, the enormous volume, variety, and velocity of HTPP data generated by such platforms make it a ‘big data’ problem. Big data generated by these near real-time platforms must be efficiently archived and retrieved for analysis [27]. The analysis and interpretation of these large datasets is quite challenging, although several authors have proposed using sensor data through a linear regression based on standard ordinary least squares [29]. To overcome collinearity among predictors (bands from the sensor), Hernandez et al. [14] concluded that penalized ridge regression models from spectral reflectance data at anthesis or grain-filling predict grain yield well under different water levels. Recently, Ferragina et al. [12] proved that high-dimensional Bayesian regression models (similar to those used in genomic selection for predicting the performance of unobserved individuals based on a large number of markers) can be used to derive functions of hundreds of wavelengths. However, no Bayesian regression models or other functional regression models that define the function of wavelength for the prediction of grain yield and other traits in different environmental conditions have been studied in plant breeding [6].

Based on the above considerations, the main objectives of this study are: (1) to compare prediction accuracy of eight conventional spectral VIs (see Table 1) versus seven models that include ordinary least squared regressions for each spectral VI and all spectral VIs combined, Bayes B with all bands, Principal Components (PC) Bayes B regression, and three functional regression models, spline regression, Fourier regression and Partial Least Regression (PLS); (2) to identify the best models in terms of prediction accuracy; and (3) to identify time-points of plant growth before harvesting from which accurate predictions for wheat grain yield can be obtained. We illustrate the use of the different methods and models with data on grain yield collected on 1170 CIMMYT wheat lines evaluated in five contrasting environments. A total of 250 wavelengths were used on nine different time data points of crop growth.

**Table 1:** Spectral reflectance indices

Index	Name	Physiological process	Type	Calculation
RNDVI	Red normalized difference vegetation index	Green area, photosynthetic capacity, N status	VI	[*x*(780) − *x*(670)]/[*x*(780) + *x*(670)]
GNDVI	Green normalized difference vegetation index	Green area, photosynthetic capacity, N status	VI	[*x*(780) − *x*(550)]/[*x*(780) + *x*(550)]/
SRa	Simple ratio	Green biomass	VI	[*x*(800)/]/[*x*(680)] and [*x*(900)]/[*x*(680)]
RARSa	Ratio analysis of reflectance spectra chlorophyll a	Chlorophyll a content	PI	[*x*(675)]/[*x*(700)]
RARSb	Ratio analysis of reflectance spectra chlorophyll b	Chlorophyll b content	PI	[*x*(675)]/[*x*(650) × *x*(700)]
RARSc	Ratio analysis of reflectance spectra chlorophyll c	Chlorophyll c content	PI	[*x*(760)]/[*x*(500)]
NPQI	Normalized pheophytinization index	Normal chlorophyll degradation; can be used to estimate phenology, pest and diseases	PI	[*x*(415) − *x*(435)]/[*x*(415) + *x*(435)]
PRI	Photochemical reflectance index	Dissipation of excess radiation	PI	[*x*(531) − *x*(570)]/[*x*(531) + *x*(570)]

Index types: *VI* vegetation index, *PI* pigmented related index [19]

## Methods

### Data

All 1170 lines were evaluated in all environments [Drought (severe drought), EarlyHeat (irrigated early planting for heat at sowing), Irrigated (irrigated bed planting), Melgas (irrigated flat planting) and Reduced Irrigated (moderate drought)]. In each environment, the lines were included in 39 trials, each comprising 30 lines; in each trial, the lines were studied using an alpha-lattice design with three replicates and six experimental blocks. The planting dates were all in 2013 as follows: EarlyHeat on October 30, Irrigated and Reduced Irrigated on November 21, Drought on November 26 and Melgas on December the 1st. The traits measured on each line were grain yield (GY) and days to heading (DH), but only GY was analyzed in this study.

The image data was obtained using a Piper PA-16 Clipper flight that was fitted with a Hyperspectral camera (Model: A-series, Micro-Hyperspec Airborne sensor, VNIR Headwall Photonics, www.headwallphotonics.com, Fitchburg, Massachusetts, US) and thermal camera (A600 series Infrared camera, FLIR, www.flir.com, Boston, US). The plane flew at 270 m above the surface.

The aerial high throughput phenotyping (HTP) data was measured around solar noon time every date, aligning the plane to the solar azimuth for the data acquisition. Images of the experimental fields were obtained and formatted to tabular data by calculating the mean value of the pixels inside the center of each individual trial plot represented as a polygon area on a map. The software used to achieve this was ArcMap (ESRI, USA, CA).

On the data processing, the 38 cm per pixel CT data was corrected with a linear calibration of slope 1.2253 and Y intercept −6767.9 with the software ImapQ (Alava Ingenieros, Madrid, Spain). The several individual images of each flight were used to compose a unique mosaic per date with the software Autopano Giga (Kolor SARL, France). Then they were manually georeferenced using ArcMap (ESRI, USA, CA). The original image data is stored in kelvin units ×100, the next formula was applied with ENVI software (Excelis VIS, USA, CO) to convert the pixel values to Celsius degrees: (Pixel value)/100 − 273.15.

As well, the 30 cm per pixel hyperspectral data was processed with the software HyproQ (Alava Ingenieros, Madrid, Spain). First the images had a radiometric calibration with coefficients provided by the Laboratory for Research Methods in Quantitative Remote Sensing of the Consejo Superior de Investigaciones Científicas (QuantaLab, IAS-CSIC, Spain) derived with a calibrated uniform light source; additionally the dark frame subtraction was performed to reduce the noise of the sensor. Corrections to decrease the effects of the atmosphere conditions in the images was performed modeling irradiance based on sun-photometer field measurements (Microtops II, Solar Light Company, PA, USA). The images were orthorectified and coarsely georeferenced based on the built-in Inertial Navigation System (INS/GPS). For the data extraction, where the image did not overlay the plots polygons because of INS inaccuracy, they were manually aligned to it in ArcMap.

The bands were measured on nine different dates (January 10, 2015; January 17, 2015; January 30, 2015; February 7, 2015; February 14, 2015; February 19, 2015; February 27, 2015; March 11, 2015; and March 17, 2015; which we called time-points 1, 2, 3,…, 9, respectively) using 250 discrete narrow wavelengths. In each plot, 250 wavelengths λ_1_, … λ_250_ from 392.03 to 850.69 nm were measured for each wheat line. The ith discretized spectrometic curve is given by x_1_(λ_1_), …, x_n_(λ_250_). We used the notation x(780) without subscripts to denote the response of the band measured at a wavelength of 780 nm, x(670) to denote the response of the band measured at a wavelength of 670 nm, and so on.

Note that early heat trial was planted in average 26 days earlier than the other trials; therefore, comparisons between individual time-points between heat trial and the others environments should consider data on the number of weeks since sowing. The comparison between the environments except early heat trial can be done more less fairly since the heading date ranged from 77 to 82 days after sowing, a period of five days in average. In all the environments heading date happened after the time-point four except in the Melgas environment in which heading date occurred at the same moment of time-point six.

### Definition and computation of spectral vegetation indices

Eight different VIs were constructed with the 250 bands and are described in Table 1.

### Statistical methods

#### Adjusting the original data set

The lines were evaluated using an alpha-lattice design with three replicates and six incomplete blocks each, with five wheat lines randomly distributed within the incomplete block. This alpha-lattice design was established for each of the five environments. First, the design effect was removed in each environment and the BLUPs (Best Linear Unbiased Predictor) of genotypes for GY, for each of the 250 wavelengths and for each of the eight VIs were obtained in each of the nine time-points using the following model

$$ y_{ijkl} = \mu + g_{i} + t_{j} + r_{k\left(j\right)} + b_{l\left({k, j}\right)} + \epsilon_{ijkl}, $$

where *μ* is the overall mean,$y_{ijkl}$ is the response variable (GY, wavelength measure and VIs) for the *i*th genotype, *j*th trial, *k*th replicate, and *l*th block, *g*_*i*_ is the random genetic effect of genotype *i* with normal distribution $N\left( {0,\upsigma_{g}^{2} } \right)$, *t*_*j*_ is the random effect of trial *j* with normal distribution $N\left( {0,\upsigma_{t}^{2} } \right)$, $r_{k\left( j \right)}$ is the random effect of replicate *k* nested within trial *j* with normal distribution $N\left( {0,\upsigma_{r}^{2} } \right)$, *b*_*l*(*k*,*j*)_ is the random effect of the incomplete block *l* nested within replicate *k* and trial *j* with normal distribution $N\left( {0,\upsigma_{b}^{2} } \right)$, and $\epsilon_{ijkl}$ is the residual effect with normal distribution $N\left( {0,\upsigma_{e}^{2} } \right)$. After these pre-adjustments in each environment, we obtained BLUPs for each of the 1170 genotypes for GY, for each of the 250 bands and for each of the eight VIs. The BLUPs of genotypes were obtained for each of the nine time-points under study. Also, from fitting the alpha-lattice experimental model expressed above, we used the variance components of genotypes and of the error term to calculate the broad-sense heritability using the expression $H^{2} = \frac{{\upsigma_{g}^{2} }}{{\upsigma_{g}^{2} +\upsigma_{e}^{2} }}$ [10]; this was calculated for each of the 250 bands in each time-point in each environment.

#### Proposed single time-point models for the adjusted data

With the pre-adjusted data (BLUPs of genotypes for GY, for each of the 250 bands and the eight VIs), we propose to evaluate prediction accuracy for each time-point using the following statistical models:

- *Model 1* Index ordinal least square (OLS) regression: $y_{i} = \mu + z_{im} \alpha_{m} + \epsilon_{i}$,
- *Model 2* Joint index OLS regression: $y_{i} = \mu + \sum \nolimits_{m = 1}^{8} z_{im} \delta_{m} + \epsilon_{i}$,
- *Model 3* All bands Bayes B regression: $y_{i} = \mu + \sum\nolimits_{k = 1}^{250} x_{ik} \beta_{k} + \epsilon_{i}$,
- *Model 4* PC Bayes B regression: $y_{i} = \mu + \sum\nolimits_{l = 1}^{NPC} PC_{il} \gamma_{k} + \epsilon_{i}$,
- *Model 5* All bands functional B-spline regression: $y_{i} = \mu + \int x_{i} \left(k\right)\beta_{1} \left(k\right) dk + \epsilon_{i}$,
- *Model 6* All bands functional Fourier regression: $y_{i} = \mu + \int x_{i} \left(k\right) \beta_{2} \left(k\right) dk + \epsilon_{i}$,
- *Model 7* All bands functional PLS regression: $y_{i} = \mu + \int x_{i} \left(k\right) \beta_{3} \left(k\right) dk + \epsilon_{i}$,

where *i* = 1, …, *n*, with $n = 1170, \,k = 1, \ldots ,K$ with $K = 250, NPC$ denotes the number of principal components (PC) used and we used 6 (5, 10, 20, 35, 45 and 55). $x_{ik}$ represents reflectance at the *k*th band collected in the *i*th genotype, $PC_{il}$ are the loadings of the *lth* PC on the *ith* genotype derived from the spectra data collected, *z*_*im*_ is the *mth* index (RNDVI, GNDVI, SRa, RARSa, RARSb, RARSc, NPQI and PRI) derived from data collected at the *ith* genotype, while *x*_*i*_(*k*) is the functional predictor collected at the *ith* genotype and its corresponding functional data set is the sample [$x_{1} \left( k \right), \ldots ,x_{n} \left( k \right)]$. The error terms $\epsilon_{i}$ were assumed to be independent with null mean and variance *σ*_*E*_^2^. *α*_*m*_, *δ*_*m*_, *β*_*k*_, *γ*_*k*_, are the regression coefficients for models 1, 2, 3 and 4, respectively, while *β*_1_(*k*), $\beta_{2} \left( k \right), \beta_{3} \left( k \right)$ are the coefficient functions for functional models 5, 6 and 7, respectively.

The three proposed functional regression models (Models 5, 6 and 7) are the most popular functional regression models, where the responses are scalars and the covariates are functions. For this reason, the response variable (*y*_*i*_) is a scalar in all the proposed models and represents grain yield (GY). Also, the difference between Models 5, 6 and 7 is the basis used for representing *β*_*o*_(*k*), with *o* = 1, 2, 3. Here a basis is understood to be a set of standard functions $(\phi_{w} )_{{w \in {\mathbb{N}}}}$ that are used to approximate any function of interest by a linear combination of a sufficiently large *r*_*w*_ of these functions [23]. In Model 6, we assumed the B-spline basis; in Model 7, we used the Fourier basis; and in Model 8, the basis is the PLS. More details on the theory behind Models 5, 6, and 7 and their basis can be found in Ramsay and Silverman [23].

The parameter estimation of Models 1 and 2 was performed using OLS and implemented in the R software with the function lm() of the library MAS, while for Models 3 and 4, a Bayesian shrinkage-variable selection procedure (called Bayes B method) using a prior with a point of mass at zero and a t-slab was implemented in the BGLR R-package [20]. The functional models (Models 5, 6 and 7) were estimated with OLS and implemented in the R-package fda.usc [11] with 21 basis. First, models were fitted to the entire data set to evaluate goodness-of-fit to the training data and were then implemented through the cross-validation described in the next section. Using these 7 models, we created 19 methods (described in Table 2) according to the type of data they were applied to. The 19 methods were implemented in each of the five environments and per time-point.

**Table 2:** Methods implemented for the analyses in each environment

Method	Data	Model	Method	Data	Model
M1	RNDVI	Model 1	M11	All bands with 5PC	Model 4
M2	GNDVI	Model 1	M12	All bands with 10PC	Model 4
M3	SRa	Model 1	M13	All bands with 20PC	Model 4
M4	RARSa	Model 1	M14	All bands with 35PC	Model 4
M5	RARSb	Model 1	M15	All bands with 45PC	Model 4
M6	RARSc	Model 1	M16	All bands with 55PC	Model 4
M7	NPQI	Model 1	M17	All bands	Model 5
M8	PRI	Model 1	M18	All bands	Model 6
M9	All indices	Model 2	M19	All bands	Model 7
M10	All bands	Model 3			

It is important to point out that methods M1–M8 used only one of the 8 VIs, M9 used all 8 VIs simultaneously, M10–M19 used all 250 bands, but methods M11–M16 used all bands to perform a principal component analysis and then used different numbers of principal components (5, 10, 20, 35, 45, 55 PCs), as shown in Table 2. Additionally, methods M17 and M18 were implemented with only those bands whose heritability is >0.5.

### Assessing prediction accuracy

For the prediction accuracies of the 19 proposed methods presented in Table 2, we implemented a ten-fold cross-validation—with 1053 (90%) lines for training and 117 (10%) for testing in each fold—that was assessed by the Pearson correlation between the observed BLUPs of GY and their predicted values using the testing data set. We reported the average of the ten-fold cross-validation of the Pearson correlation (APC) as measure of prediction accuracy as well as the quantiles 2.5 (LL) and 97.5% (UL) (see “Appendices 1, 2”). It is important to point out that we used the same Split (of the ten-fold cross-validation) in the 19 methods to ensure fair comparisons between methods.

## Results

The results are given in two sections: the first section presents the heritability estimates of each of the 250 wavelengths for each environment, while the second section presents the prediction accuracies estimated under the implemented methods.

### Heritability estimates

**Fig. 1:**
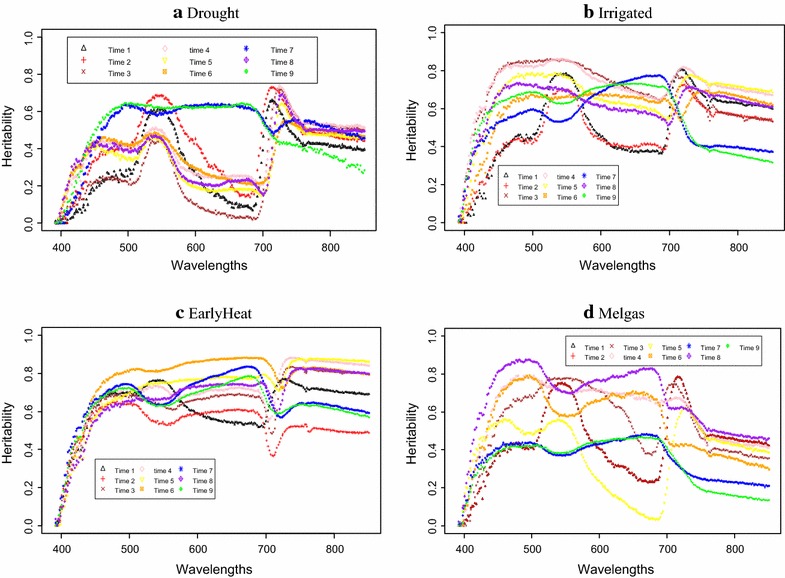
Heritability for each wavelength for environments: **a** Drought, **b** Irrigated, **c** EarlyHeat and **d** Melgas

The highest heritability estimates were found in the Irrigated (Fig. 1b) and EarlyHeat (Fig. 1c) environments, with values between 0.6 and 0.8 for most of the time-points. In these environments, heritability estimates are quite homogeneous across wavelengths, although in Irrigated, the lowest heritabilities were higher than 0.4 and were observed for wavelengths before 570 nm and those in the 580–700 nm range, while in EarlyHeat, the wavelengths with the lowest heritability were found before wavelengths of 480 nm and between wavelengths of 680–730 nm and all were higher than 0.4. On the other hand, the environment with the lowest heritability was Drought (Fig. 1b); the heritability before 450 nm and those in the 600–700 nm range are very low (around 0.2), while the rest of the bands with the highest heritability show values of around 0.6. The rest of the environments [Melgas (Fig. 1d) and Reduced Irrigated (Fig. 9; “Appendix 3”)] have intermediate heritability although they are very heterogeneous between time-points and across bands. For example, in the Melgas environment, we observed heterogeneity of heritabilities between time-points and across wavelengths and for wavelengths >750 nm for all time-points. While in Reduced Irrigated, the lowest heritabities (around 0.3) were observed in the 590–700 nm wavelength range, for six time-points (Fig. 9; “Appendix 3”).

In Early heat all time-points correspond to after heading stage while in the other trials time-points one to four were taken before heading, time points five and six during heading stage and after heading seven to nine time points. There is not a clear relationship between the heritability and the stage of the crop in which the images were taken.

### Prediction accuracies of the proposed methods

#### Comparing vegetation indices versus all bands

**Fig. 2:**
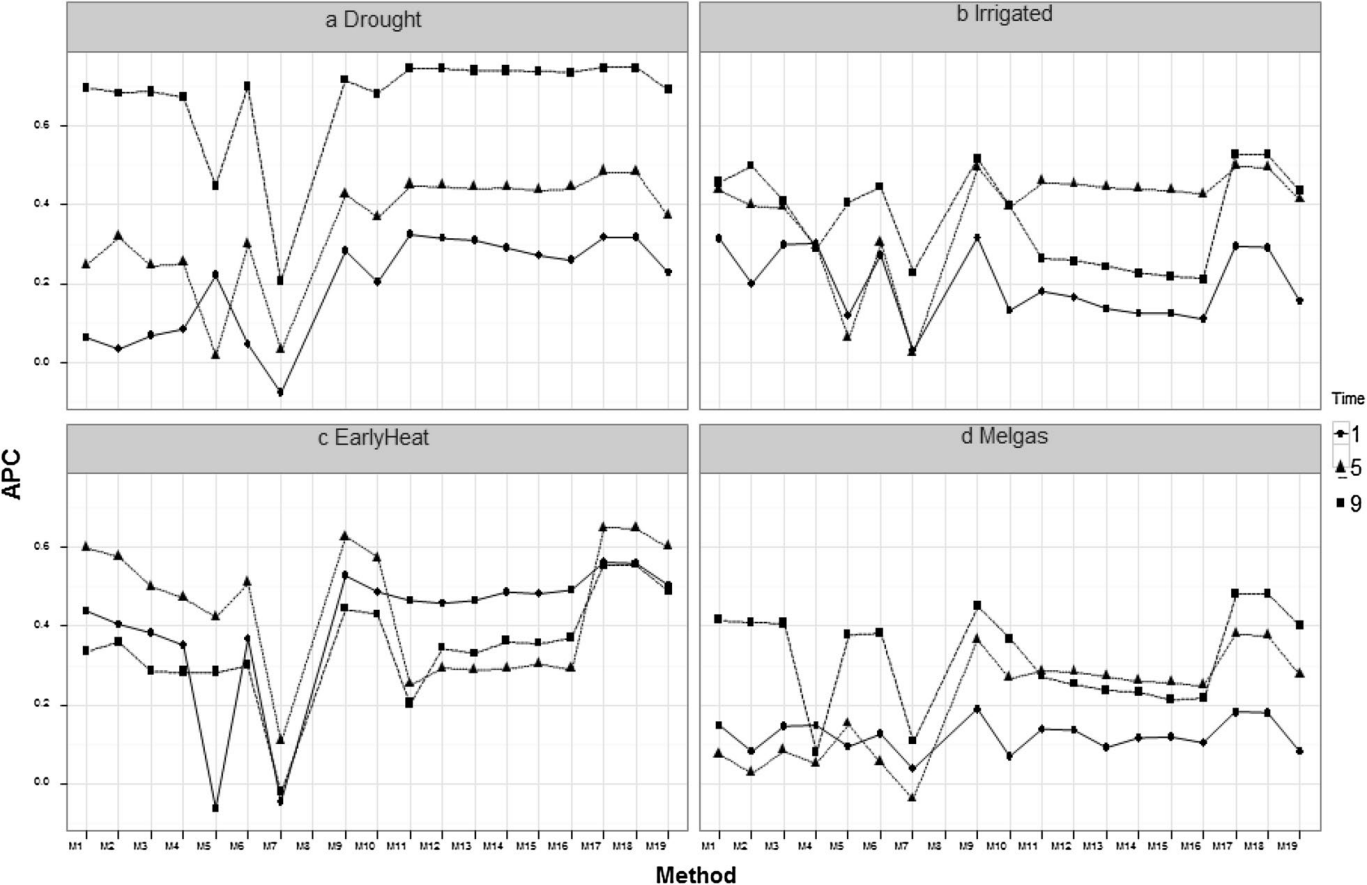
Comparing methods that use vegetation indices and those that use all bands for four environments: **a** Drought, **b** Irrigated, **c** EarlyHeat and **d** Melgas. APC means Average Pearson correlation as a measure of prediction accuracy

Figure 2 shows the prediction accuracy of Methods 1–19 in the four environments for three time-points (1, 5 and 9). In the Drought environment, the methods with the best prediction accuracy were those that used all the bands (M10–M19), while in the Irrigated environment in time-point 9, most of the methods that use all bands were the best in terms of prediction accuracy (methods M11–M19); however, in time-point 5, only methods M17 and M18 were better in terms of prediction accuracy than the methods that were built using the vegetation indices (M1–M9), but in time-point 1, methods M1, M3 and M4 built using the vegetation indices had the best prediction accuracy. In the EarlyHeat environment (Fig. 2c), the methods with the best prediction accuracy were methods M17 and M18, which use all the available wavelengths, although it is important to point out that time-point 1 was better than time-points 5 and 9 in methods M10 to M16, which use all the bands. Also, in the Melgas (Fig. 2d) and Reduced Irrigated environments (Fig. 10; “Appendix 3”), methods M17 and M18 had the best prediction accuracy. However, in these environments the best prediction was observed in time-point 9 and the worst, in time-point 1. Appendices 1 and 2 show the rest of the prediction accuracies for time-points 2, 3, 4, 6, 7 and 8 for all methods.

#### Comparing some methods for all time-points

Compared in this section are methods M1, M2, M9, M10, M17, M18, and M19. Methods M1 and M2 were chosen because they were built using two of the most widely used vegetation indices (RNDVI, GNDVI), whereas M9 uses all 8 VI simultaneously; M10 uses all bands and Bayes B. Methods M17 and M18 were included because they provided the best prediction accuracies in all the environments using all bands, while method M19 was used because it performed well in the last section using all bands.

**Fig. 3:**
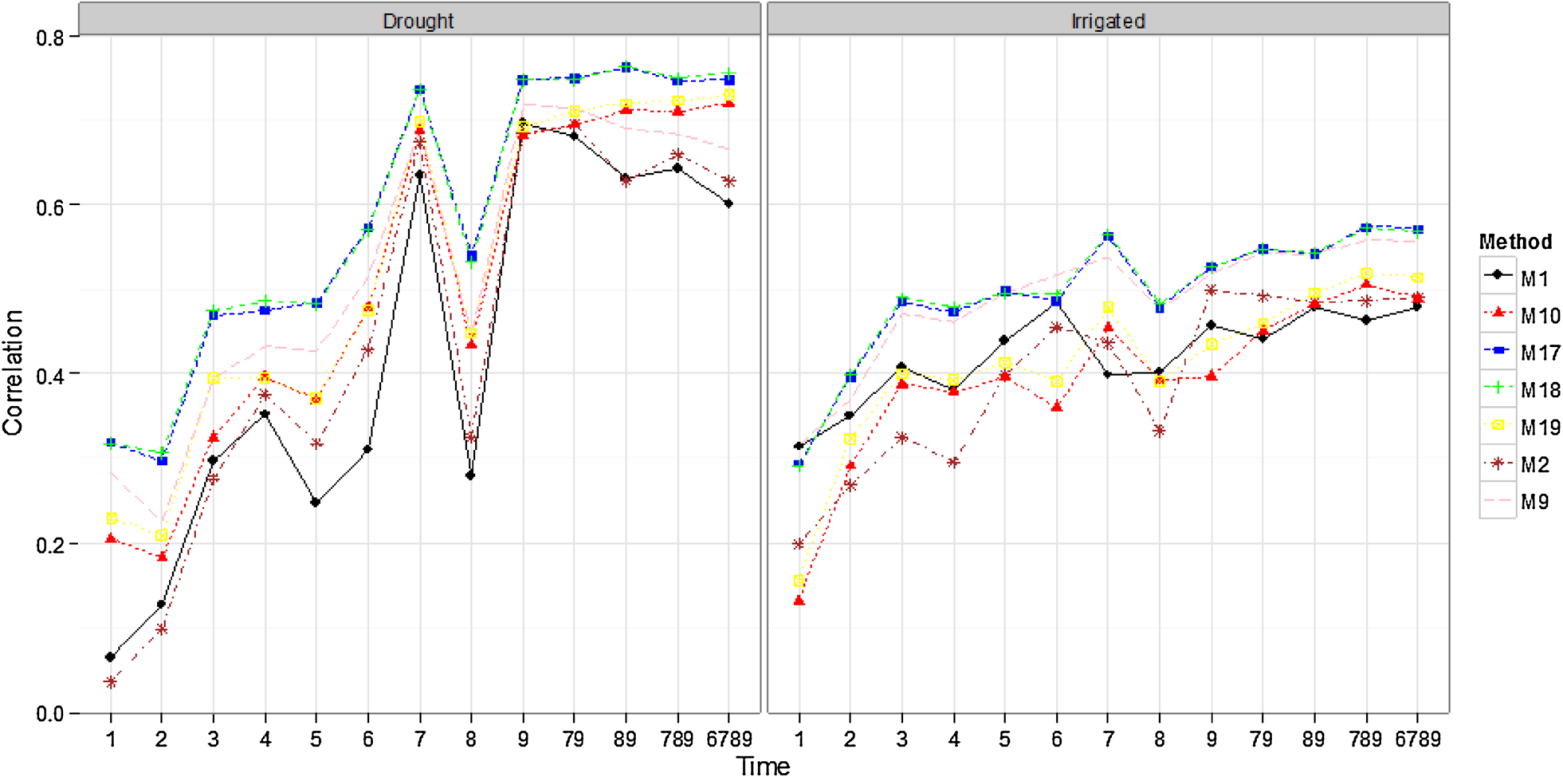
Comparison of some methods with all time-points for the Drought and Irrigated environments

**Fig. 4:**
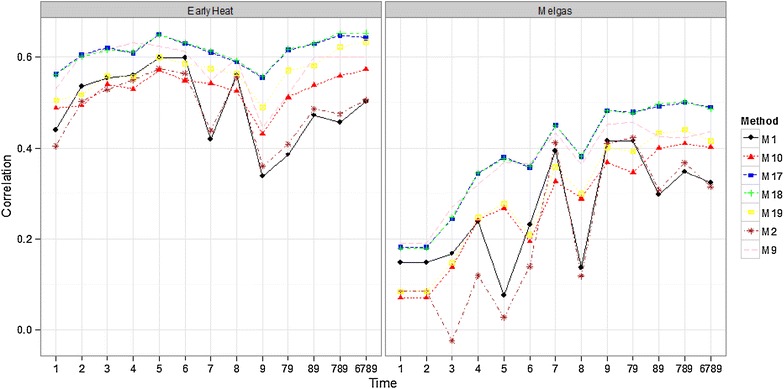
Comparison of some methods with all time-points for the EarlyHeat and Melgas environments

Drought (Fig. 3), Melgas (Fig. 4) and Reduced Irrigated (Fig. 11; “Appendix 3”) environments show that time-points below 6 had lower prediction accuracies and the best predictions were from points 7, 9 and the joint time-points 79, 89, 789, and 6789. Time-point 79, 89, 789 and 6789 were obtained as the average of the time-points 7 and 9, 8 and 9, 7, 8 and 9, and 6, 7, 8 and 9 respectively; this nomenclature is used in the rest of the manuscript. It is important to point out that the prediction accuracies of time-point 8 were considerably lower than those of time-points 7, 9, and 6. In the Irrigated environment (Fig. 3), a similar trend is observed, yet for some methods (M17 and M18), time-point 7 provides the best predictions. In this environment (Irrigated), the differences in prediction accuracy between time-point 5 and time-points 6, 7, 8, 9, 79, 89, 789, and 6789 are not strong. This indicates that even with time-point 5, we can generate good prediction accuracies for grain yield. In the EarlyHeat environment (Fig. 4), all time-points produced good predictions, although methods M1 and M2 produced lower predictions in time-points 7, 9, 79, 89, 789 and 6789. It is important to point out that methods M17 and M18 were the best in all time-points in all environments, although in environments EarlyHeat and Melgas, the superiority of these methods is clearer.

#### Comparing environments for time-points 5 and 9

**Fig. 5:**
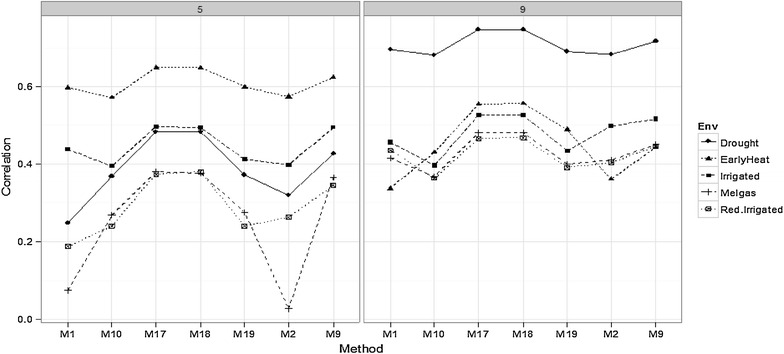
Comparison of environments for some methods in time-points *5* and *9*

**Fig. 6:**
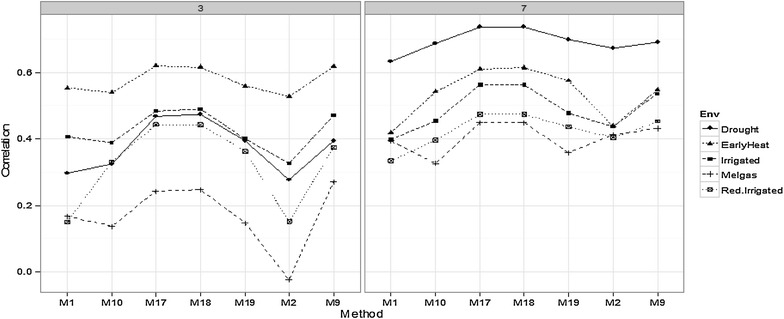
Comparison of environments for some methods in time-points *3* and *7*

Figures 5 and 6 show that there are differences in prediction accuracy between environments. In time-point 5 (Fig. 5), EarlyHeat was the environment with the best predictions, followed by Irrigated and Drought, while the worst predictions were observed in Melgas and Reduced Irrigation. In time-point 3 (Fig. 6), the behavior was similar to that of time-point 5, since EarlyHeat was also the best in terms of prediction accuracy; however, here Melgas was the worst and the other three environments were in the middle. In time-points 7 (Fig. 6) and 9 (Fig. 5), the pattern was different since here the best predictions were in the Drought environment, and the second best was EarlyHeat, since in four of the seven methods presented in Fig. 5, this environment had the second best predictions. In third place is the Irrigated environment, while the worst predictions were observed in Melgas and Reduced Irrigated. It is important to point out that methods M17 and M18 were consistently the best in the five environments. Furthermore, it should be noted that the planting date in EarlyHeat is around 5 weeks earlier than the planting dates in the other four environments; thus the comparison of prediction performance at the same time-points does not represent a comparison at the same crop development stage.

#### Comparing methods M17 and M18 using all the bands and bands with heritabilities >0.5

**Fig. 7:**
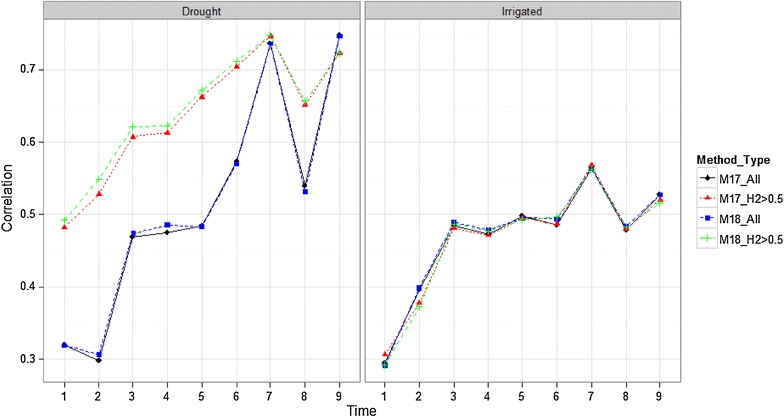
Comparison of methods M17 and M18 with all bands and with bands with heritability >0.5 for all time-points in environments Drought and Irrigated

Figure 7 compares methods M17 and M18 for all time points using all bands and only those bands with heritabilities >0.5. We observe in Drought that when only the bands with heritabilities >0.5 were used, prediction accuracies were better than when using all bands for both methods (M17 and M18). However, in the Irrigated environment using all bands, prediction accuracies were slightly better than when using only the bands with heritability >0.5; however, the difference is not relevant.

**Fig. 8:**
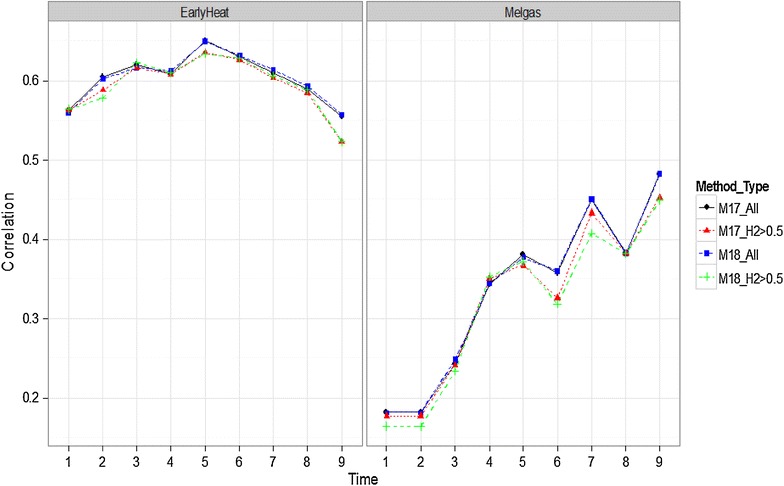
Comparison of methods M17 and M18 with all bands and with bands with heritability >0.5 for all time-points in environments EarlyHeat and Melgas

**Fig. 9:**
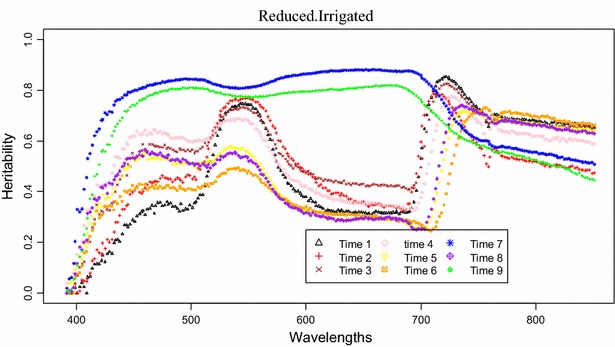
Heritability of each wavelength for the Reduced Irrigated environment

**Fig. 10:**
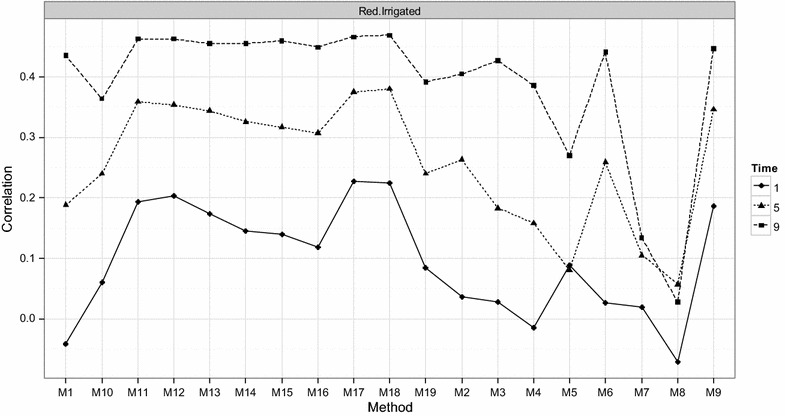
Comparison of methods that use vegetation indices and those that use all bands for the Reduced Irrigated environment

**Fig. 11:**
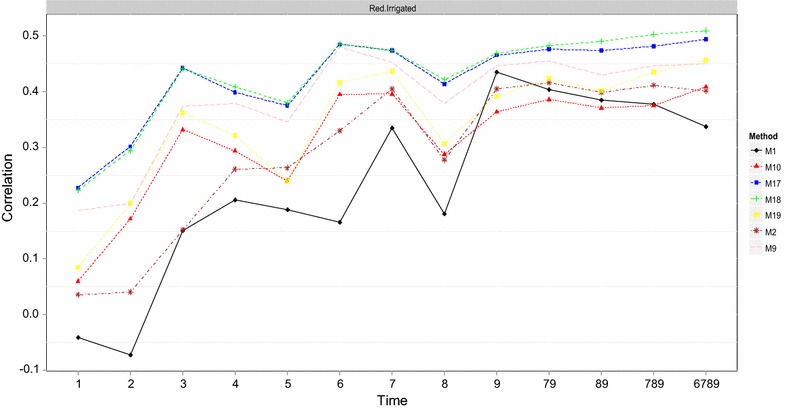
Comparison of some methods with all time-points for the Reduced Irrigated environment

**Fig. 12:**
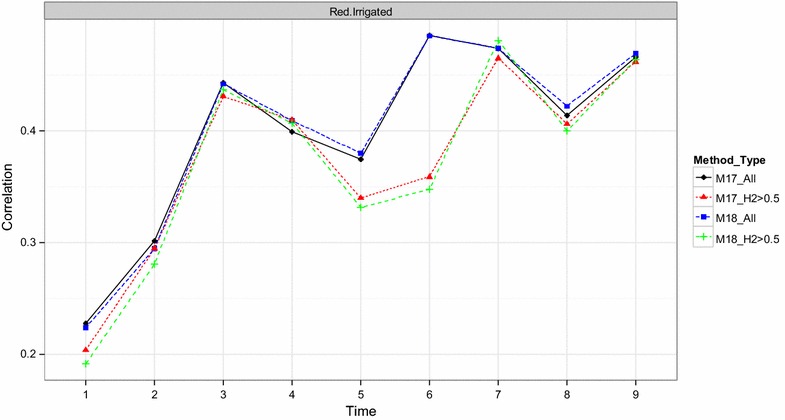
Comparison of methods M17 and M18 with all bands and with bands with heritability >0.5 for all time-points in the Reduced Irrigated environment

In Fig. 8, we observe that in both environments (EarlyHeat and Melgas), using all the bands was a little better than using only the bands with heritabilities >0.5, although the differences were not significant. The same pattern is observed for the Reduced Irrigated environment (Fig. 12; “Appendix 3”).

## Discussion

### Heritability estimates of the bands

Results indicated that the heritabilities of each wavelength are not homogeneous across groups. The Irrigated and EarlyHeat environments had the highest heritabilities (with values between 0.6 and 0.8), which were homogenous across wavelengths, while Drought had the lowest heritabilities (with lowest values around 0.2), which were heterogeneous across wavelengths and time-points. Results in “Comparing vegetation indices vs all bands” section indicate that using all bands simultaneously as explanatory variables produced better prediction accuracies that using the VIs alone or combined. However, predictions were better when using only those bands with heritabilities >0.5 compared with using all bands only in Drought, while in the other 4 environments, using all bands produced slightly better prediction accuracies (“Comparing methods M17 and M18 using all the bands and bands with heritabilities>0.5” section). Therefore, the evidence indicates that using all bands simultaneously provided better prediction accuracies than using the VI alone or combined and even than using those bands with heritabilities >0.5. However, it is important to point out that the methods that used VI alone as predictor variables are very heterogeneous in terms of prediction accuracy, since some performed very poorly, while others produced reasonable predictions (for example, M6).

### Prediction accuracy of the methods

Since we now have enough evidence to say that using all bands produced better predictions than using individual, combined VI and even when we restrict the models to less noisy features (H2 > 0.5), we compared the methods that used all the bands. Based on the prediction accuracy of the methods, results indicate that for this data set, methods M17 and M18 are the best for prediction. These two methods were better in all environments and in most of the nine time-points, and were also considerably better than the PC methods (M11 to M16), the Bayes B method (M10), and a little better than the functional PLS method (M19). The best two methods (M17 and M18) are functional regression models and correspond to models 5 and 6 described in “Proposed single time-point models for the adjusted data” section. Functional regression models nowadays have become an increasingly important statistical tool when the number of covariates is larger than the number of observations, where the unit of observation is generally viewed as a function or a curve defined based on some underlying continuous domain, and the observed data consist of a sample of functions taken from some population, sampled on a discrete grid.

Given the nature of our data, the functional regression that we implemented only considered functional predictors; however, this regression method can also be used when both the predictors and the responses are functions. For this reason, functional regression models have been implemented successfully in many research areas (spectroscopy, economics, environmental studies, bioscience, system engineering, etc.). Functional regression is also very attractive because it is a non-destructive technology that measures numerous chemical compounds in a variety of products (plant, soil, food, petroleum, wood products, etc.) and can be used in large databases in experimental and non-experimental settings.

### Prediction accuracy for time-points

Regarding the prediction accuracy for time-points, in general, prediction accuracies before time-point 6 were poor in four environments, and all time-points produced good predictions only in the EarlyHeat environment; a likely explanation for this may be that in this environment the sowing date was around 5 weeks earlier than the sowing dates in the other 4 environments, that is, the development of the crop for all time-points was more advanced in EarlyHeat. For this reason, the empirical evidence indicates that, for this dataset, time-point 6 achieved good prediction accuracy. Also, in general, time-point 6 predictions are better than time-point 8 predictions. However, we need to be careful when interpreting time-point 6, since sowing time was different in each environment and the plants were at different growth stages when the bands were measured. Using this time-point can be helpful for breeders, since it is around 28 days before time-point 9. Also, it is important to point out that the predictions of the average time-points under study (79, 89, 789, and 6789) are a little better than those of time-points 6, 7 and 9 in methods that used all the bands; however, the increase in prediction accuracy is not large.

## Conclusions

In this research, we proposed using all the bands simultaneously as predictor variables instead of using only one VI alone or all the VI together. First, we found that the heritabilities of the bands were heterogeneous across time-points and environments and that the best heritabilities were observed in the Irrigated and EarlyHeat environments and the worst in the Drought environment. We found that using all the bands simultaneously produced better predictions than using one VI alone or all the VI together. When we used only the less noisy bands (H2 > 0.5) in Drought, the predictions improved, while in the rest of the environments the results were similar. Out of the methods that used all the bands, the best methods across time-points and environments were M17 and M18 (functional B-spline and Fourier, respectively). Also, time-point 6 and 8 predictions were slightly lower than those of time-points 7 and 9, yet were close enough to be used for the prediction of wheat lines before harvesting. Finally, results show that the approach used to analyze high-resolution image data used in this study is promising; however, it is also clear that its application in this context is not straightforward, since the Bayes B method, which is popular for genomic selection, did not produce the best predictions. There are many challenges that need to be considered in future research using functional regression models, such as the inclusion of genotype × environment interaction, random effects, traits not normally distributed and multiple traits as response variables. Also, other conventional methods (GBLUP, Bayes A, Ridge Regression, Bayes C) used in genomic-enabled prediction should be tested in the context of high-resolution imaging data.

## Figures and Tables

**Fig. 13:**
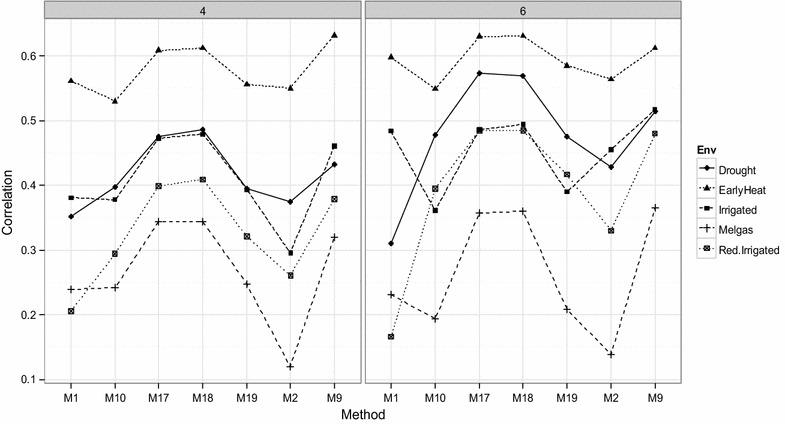
Comparison of environments for some methods in time-points *4* and *6*

**Fig. 14:**
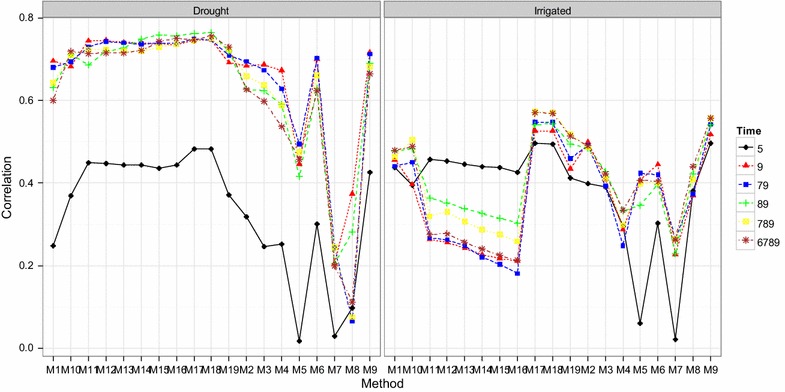
Comparison of all methods for time-points *5*, *9*, *79*, *89*, *789*, *6789* in Drought and Irrigated environments

**Fig. 15:**
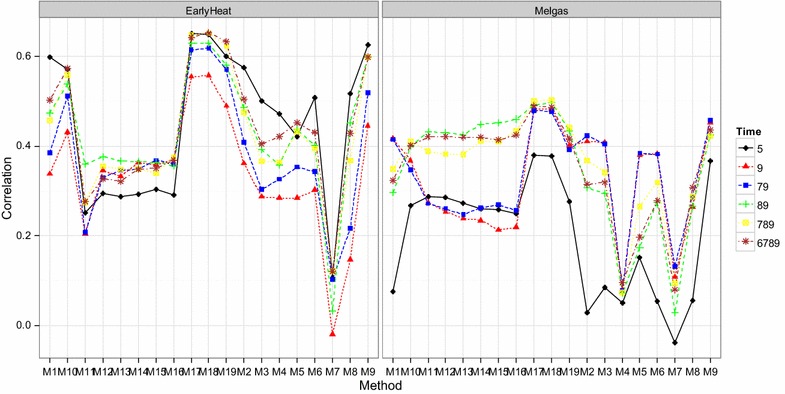
Comparison of all methods for time-points *5*, *9*, *79*, *89*, *789*, *6789* in EarlyHeat and Melgas environments

**Fig. 16:**
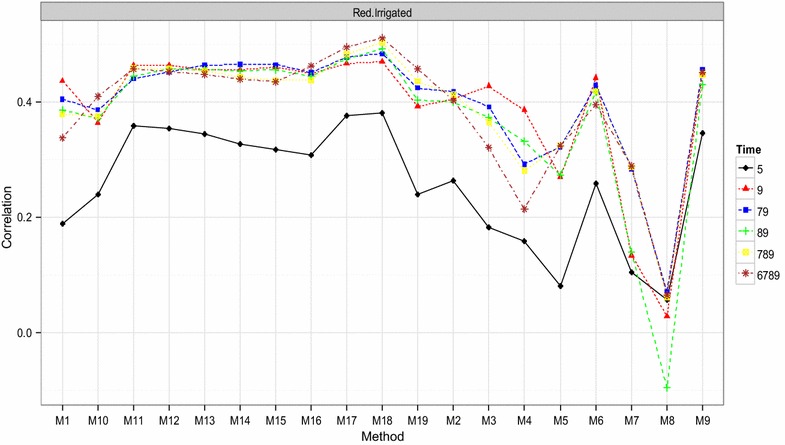
Comparison of all methods for time-points *5*, *9*, *79*, *89*, *789*, *6789* in EarlyHeat and Melgas environments

## Appendix 1

See Table 3.

**Table 3:** Average Pearson correlation (APC), quantile 2.5% (LL) and quantile 97.5% (UL) for the testing set between the observed GY and the predicted GY using only the vegetation index as the predictor variable in each of the environments

Method	Time	Melgas			Drought			Irrigated			Red.Irrigated			EarlyHeat		
		APC	LL	UL	APC	LL	UL	APC	LL	UL	APC	LL	UL	APC	LL	UL
M1	1	0.148	−0.024	0.312	0.065	−0.103	0.302	0.314	0.173	0.450	−0.041	−0.148	0.072	0.440	0.328	0.566
M1	2	0.148	−0.024	0.312	0.127	−0.028	0.284	0.350	0.267	0.476	−0.072	−0.207	0.010	0.535	0.453	0.623
M1	3	0.167	−0.016	0.280	0.297	0.183	0.434	0.407	0.241	0.525	0.151	0.075	0.254	0.553	0.435	0.667
M1	4	0.239	0.043	0.401	0.352	0.242	0.543	0.381	0.189	0.489	0.206	0.089	0.313	0.561	0.434	0.669
M1	5	0.075	−0.054	0.226	0.248	0.143	0.427	0.438	0.194	0.542	0.188	0.027	0.279	0.598	0.497	0.693
M1	6	0.231	0.025	0.407	0.310	0.191	0.460	0.484	0.359	0.577	0.166	−0.013	0.278	0.598	0.470	0.691
M1	7	0.394	0.275	0.475	0.634	0.509	0.762	0.398	0.189	0.507	0.335	0.196	0.465	0.419	0.330	0.524
M1	8	0.137	−0.091	0.297	0.280	0.174	0.464	0.403	0.239	0.497	0.181	−0.017	0.267	0.560	0.438	0.679
M1	9	0.416	0.312	0.491	0.696	0.616	0.814	0.457	0.336	0.542	0.436	0.286	0.610	0.338	0.255	0.431
M1	6789	0.324	0.178	0.439	0.601	0.486	0.740	0.479	0.318	0.574	0.337	0.169	0.481	0.503	0.411	0.612
M1	789	0.349	0.229	0.451	0.643	0.538	0.774	0.463	0.289	0.563	0.378	0.215	0.527	0.457	0.376	0.569
M1	79	0.415	0.301	0.481	0.681	0.584	0.803	0.441	0.272	0.536	0.404	0.266	0.565	0.385	0.310	0.486
M1	89	0.297	0.133	0.416	0.632	0.540	0.776	0.478	0.328	0.571	0.385	0.202	0.537	0.473	0.399	0.585
M2	1	0.084	−0.069	0.246	0.036	−0.116	0.244	0.199	0.015	0.327	0.036	−0.085	0.248	0.405	0.286	0.579
M2	2	0.084	−0.069	0.246	0.099	−0.100	0.280	0.269	0.113	0.412	0.040	−0.207	0.196	0.502	0.404	0.605
M2	3	−0.023	−0.171	0.114	0.277	0.178	0.372	0.326	0.170	0.453	0.152	0.065	0.283	0.528	0.393	0.651
M2	4	0.120	−0.053	0.324	0.375	0.281	0.536	0.296	0.145	0.392	0.261	0.114	0.388	0.550	0.411	0.689
M2	5	0.028	−0.149	0.192	0.319	0.162	0.510	0.399	0.188	0.502	0.263	0.133	0.334	0.575	0.454	0.675
M2	6	0.139	−0.013	0.297	0.428	0.319	0.586	0.455	0.340	0.547	0.330	0.215	0.398	0.564	0.430	0.653
M2	7	0.412	0.316	0.474	0.673	0.592	0.779	0.437	0.275	0.537	0.405	0.288	0.532	0.439	0.377	0.530
M2	8	0.119	−0.064	0.293	0.325	0.220	0.513	0.333	0.198	0.435	0.278	0.099	0.371	0.556	0.420	0.666
M2	9	0.410	0.303	0.492	0.684	0.611	0.788	0.499	0.369	0.587	0.405	0.311	0.559	0.361	0.257	0.445
M2	6789	0.315	0.219	0.424	0.627	0.544	0.753	0.490	0.361	0.570	0.402	0.281	0.498	0.505	0.414	0.604
M2	789	0.368	0.292	0.475	0.659	0.581	0.778	0.486	0.351	0.579	0.412	0.286	0.525	0.475	0.400	0.575
M2	79	0.423	0.317	0.491	0.695	0.622	0.799	0.491	0.372	0.587	0.417	0.308	0.556	0.408	0.329	0.494
M2	89	0.307	0.196	0.443	0.628	0.545	0.757	0.485	0.359	0.576	0.399	0.275	0.512	0.486	0.407	0.589
M3	1	0.146	−0.039	0.309	0.070	−0.079	0.290	0.300	0.158	0.433	0.028	−0.100	0.192	0.384	0.274	0.514
M3	2	0.146	−0.039	0.309	0.139	−0.010	0.308	0.334	0.260	0.461	−0.034	−0.206	0.058	0.445	0.353	0.518
M3	3	0.162	−0.045	0.301	0.321	0.200	0.485	0.362	0.208	0.480	0.162	0.061	0.277	0.437	0.343	0.544
M3	4	0.225	0.065	0.390	0.380	0.269	0.596	0.339	0.167	0.441	0.221	0.077	0.339	0.454	0.355	0.554
M3	5	0.084	−0.112	0.246	0.247	0.119	0.453	0.392	0.183	0.493	0.183	0.008	0.308	0.500	0.428	0.576
M3	6	0.225	0.053	0.380	0.300	0.162	0.463	0.420	0.300	0.509	0.151	−0.034	0.272	0.501	0.429	0.577
M3	7	0.381	0.276	0.474	0.629	0.500	0.765	0.352	0.158	0.447	0.311	0.134	0.476	0.313	0.216	0.440
M3	8	0.138	−0.088	0.326	0.286	0.182	0.511	0.358	0.201	0.446	0.178	−0.012	0.274	0.475	0.384	0.555
M3	9	0.408	0.288	0.479	0.687	0.596	0.805	0.409	0.313	0.482	0.427	0.290	0.631	0.287	0.198	0.394
M3	6789	0.319	0.183	0.428	0.599	0.487	0.738	0.423	0.270	0.496	0.321	0.147	0.495	0.405	0.338	0.504
M3	789	0.342	0.220	0.447	0.637	0.533	0.766	0.412	0.250	0.488	0.365	0.197	0.549	0.366	0.293	0.472
M3	79	0.405	0.290	0.490	0.674	0.572	0.799	0.393	0.244	0.467	0.391	0.253	0.591	0.304	0.222	0.418
M3	89	0.294	0.135	0.423	0.624	0.536	0.766	0.428	0.295	0.497	0.373	0.196	0.555	0.393	0.335	0.483
M4	1	0.149	−0.035	0.340	0.086	−0.055	0.272	0.303	0.175	0.446	−0.014	−0.154	0.082	0.351	0.215	0.467
M4	2	0.149	−0.035	0.340	0.094	−0.059	0.200	0.327	0.219	0.456	0.039	−0.168	0.157	0.437	0.318	0.515
M4	3	0.224	0.094	0.347	0.312	0.170	0.414	0.311	0.110	0.408	0.194	0.048	0.280	0.446	0.328	0.566
M4	4	0.190	0.039	0.330	0.362	0.222	0.576	0.297	0.032	0.430	0.209	0.060	0.322	0.412	0.298	0.502
M4	5	0.051	−0.111	0.183	0.252	0.115	0.467	0.296	0.010	0.472	0.158	−0.005	0.265	0.471	0.387	0.584
M4	6	0.124	−0.076	0.271	0.148	−0.027	0.315	0.372	0.224	0.497	0.021	−0.075	0.211	0.547	0.391	0.675
M4	7	0.069	−0.046	0.187	0.530	0.381	0.669	0.199	−0.048	0.329	0.131	−0.026	0.230	0.356	0.242	0.491
M4	8	0.060	−0.117	0.200	0.324	0.205	0.527	0.292	0.086	0.432	0.143	−0.051	0.264	0.403	0.299	0.520
M4	9	0.080	−0.016	0.185	0.673	0.599	0.788	0.288	0.108	0.381	0.386	0.231	0.490	0.284	0.189	0.383
M4	6789	0.095	−0.065	0.234	0.537	0.412	0.699	0.335	0.129	0.438	0.214	0.021	0.335	0.421	0.322	0.552
M4	789	0.074	−0.056	0.202	0.589	0.476	0.740	0.298	0.073	0.405	0.281	0.108	0.403	0.364	0.260	0.485
M4	79	0.077	−0.031	0.175	0.629	0.517	0.762	0.248	0.028	0.357	0.292	0.149	0.391	0.326	0.225	0.443
M4	89	0.072	−0.085	0.195	0.590	0.502	0.745	0.332	0.133	0.435	0.331	0.127	0.466	0.358	0.264	0.468
M5	1	0.096	−0.019	0.189	0.223	0.132	0.350	0.120	0.046	0.194	0.089	−0.138	0.301	−0.062	−0.217	0.061
M5	2	0.096	−0.019	0.189	0.130	−0.033	0.249	0.086	−0.007	0.182	0.170	0.019	0.361	0.296	0.151	0.392
M5	3	0.232	0.063	0.356	0.118	−0.057	0.225	0.050	−0.131	0.184	0.119	−0.061	0.267	0.368	0.267	0.474
M5	4	0.113	−0.083	0.222	0.085	−0.006	0.181	0.033	−0.101	0.127	0.045	−0.067	0.222	0.315	0.119	0.435
M5	5	0.152	−0.008	0.308	0.018	−0.133	0.131	0.061	−0.062	0.162	0.080	−0.014	0.254	0.422	0.267	0.500
M5	6	0.055	−0.073	0.147	0.174	0.016	0.280	0.249	0.106	0.339	0.191	0.052	0.272	0.402	0.287	0.497
M5	7	0.371	0.260	0.554	0.482	0.346	0.596	0.423	0.371	0.538	0.351	0.197	0.474	0.393	0.300	0.537
M5	8	0.066	−0.103	0.224	−0.052	−0.134	0.024	0.012	−0.079	0.125	0.101	−0.053	0.277	0.432	0.252	0.532
M5	9	0.380	0.267	0.524	0.447	0.254	0.562	0.406	0.340	0.538	0.270	0.124	0.412	0.284	0.113	0.468
M5	6789	0.197	0.098	0.314	0.458	0.278	0.585	0.406	0.339	0.500	0.324	0.168	0.430	0.452	0.366	0.601
M5	789	0.266	0.159	0.407	0.475	0.298	0.602	0.398	0.336	0.520	0.324	0.169	0.443	0.432	0.329	0.590
M5	79	0.384	0.271	0.549	0.495	0.324	0.615	0.425	0.372	0.554	0.322	0.177	0.456	0.353	0.226	0.521
M5	89	0.174	0.051	0.288	0.417	0.215	0.544	0.346	0.269	0.457	0.273	0.133	0.426	0.437	0.320	0.595
M6	1	0.128	−0.064	0.323	0.048	−0.110	0.326	0.273	0.151	0.420	0.027	−0.092	0.177	0.370	0.232	0.521
M6	2	0.128	−0.064	0.323	0.083	−0.082	0.257	0.339	0.254	0.465	−0.036	−0.209	0.058	0.423	0.319	0.509
M6	3	0.123	−0.041	0.222	0.344	0.274	0.472	0.298	0.086	0.426	0.223	0.134	0.315	0.425	0.298	0.522
M6	4	0.206	0.010	0.366	0.392	0.305	0.608	0.255	0.044	0.381	0.287	0.138	0.396	0.437	0.297	0.556
M6	5	0.053	−0.177	0.226	0.302	0.144	0.542	0.304	0.072	0.414	0.259	0.114	0.352	0.508	0.400	0.592
M6	6	0.150	−0.056	0.294	0.383	0.270	0.557	0.386	0.234	0.478	0.271	0.131	0.363	0.515	0.410	0.598
M6	7	0.359	0.292	0.419	0.670	0.569	0.778	0.363	0.179	0.468	0.388	0.261	0.527	0.373	0.288	0.486
M6	8	0.135	−0.077	0.304	0.337	0.246	0.574	0.276	0.091	0.412	0.271	0.105	0.359	0.471	0.348	0.573
M6	9	0.383	0.279	0.451	0.701	0.613	0.811	0.446	0.319	0.522	0.441	0.349	0.618	0.302	0.207	0.403
M6	6789	0.278	0.129	0.388	0.624	0.522	0.761	0.404	0.234	0.492	0.395	0.259	0.525	0.431	0.363	0.532
M6	789	0.319	0.202	0.409	0.661	0.567	0.788	0.400	0.223	0.487	0.418	0.287	0.564	0.395	0.328	0.499
M6	79	0.382	0.294	0.432	0.703	0.611	0.811	0.420	0.275	0.508	0.428	0.314	0.595	0.343	0.256	0.447
M6	89	0.274	0.127	0.397	0.630	0.540	0.773	0.397	0.234	0.493	0.417	0.283	0.559	0.401	0.338	0.498
M7	1	0.040	−0.151	0.201	−0.077	−0.155	0.021	0.031	−0.046	0.151	0.019	−0.133	0.118	−0.046	−0.145	0.056
M7	2	0.040	−0.151	0.201	0.045	−0.082	0.258	0.030	−0.045	0.016	0.030	−0.101	0.169	−0.055	−0.139	0.013
M7	3	0.080	−0.021	0.239	0.040	−0.071	0.230	0.007	−0.105	0.074	−0.065	−0.144	0.018	0.085	−0.051	0.188
M7	4	0.030	−0.055	0.192	0.028	−0.060	0.128	0.021	−0.169	0.173	0.035	−0.098	0.176	0.039	−0.129	0.180
M7	5	−0.038	−0.183	0.049	0.030	−0.005	0.123	0.022	−0.160	0.165	0.105	−0.062	0.266	0.107	−0.008	0.257
M7	6	0.023	−0.128	0.175	−0.099	−0.196	−0.007	−0.054	−0.176	0.066	0.048	−0.016	0.163	0.100	−0.008	0.026
M7	7	0.105	−0.057	0.226	0.188	0.083	0.268	0.183	0.025	0.346	0.290	0.124	0.478	0.161	0.036	0.255
M7	8	0.027	−0.077	0.196	−0.058	−0.099	−0.016	0.170	0.020	0.345	0.055	−0.065	0.138	0.106	0.032	0.202
M7	9	0.108	−0.013	0.207	0.206	0.080	0.315	0.228	0.107	0.308	0.134	−0.004	0.285	−0.020	−0.151	0.084
M7	6789	0.080	−0.044	0.167	0.200	0.075	0.294	0.263	0.170	0.391	0.288	0.158	0.439	0.121	−0.018	0.239
M7	789	0.094	−0.069	0.195	0.244	0.137	0.325	0.264	0.169	0.391	0.286	0.152	0.440	0.121	−0.018	0.239
M7	79	0.132	−0.027	0.251	0.244	0.137	0.325	0.264	0.169	0.391	0.283	0.147	0.434	0.103	−0.029	0.222
M7	89	0.029	−0.114	0.115	0.206	0.080	0.315	0.228	0.107	0.308	0.140	−0.011	0.299	0.032	−0.059	0.178
M8	1	0.126	0.059	0.251	0.019	−0.121	0.099	0.255	0.114	0.351	−0.071	−0.126	−0.019	0.376	0.284	0.521
M8	2	0.126	0.059	0.251	0.074	−0.067	0.157	0.238	0.156	0.326	−0.080	−0.176	0.012	0.519	0.449	0.583
M8	3	−0.050	−0.155	0.012	0.146	0.055	0.252	0.364	0.228	0.500	−0.016	−0.118	0.059	0.511	0.434	0.588
M8	4	0.022	−0.119	0.209	0.176	0.079	0.328	0.372	0.288	0.465	0.050	−0.077	0.184	0.517	0.437	0.613
M8	5	0.056	−0.013	0.196	0.098	0.009	0.232	0.381	0.183	0.507	0.057	−0.087	0.229	0.517	0.448	0.625
M8	6	0.210	0.072	0.389	0.133	−0.045	0.206	0.434	0.357	0.531	0.053	−0.197	0.200	0.486	0.423	0.571
M8	7	0.227	0.052	0.403	0.277	−0.010	0.448	0.327	0.122	0.455	0.104	−0.049	0.205	0.216	0.124	0.321
M8	8	0.038	−0.067	0.147	0.046	−0.087	0.156	0.379	0.323	0.438	−0.071	−0.179	−0.013	0.506	0.407	0.617
M8	9	0.264	0.089	0.346	0.374	0.213	0.469	0.371	0.267	0.467	0.028	−0.118	0.287	0.147	0.039	0.246
M8	6789	0.308	0.112	0.415	0.111	−0.190	0.276	0.441	0.334	0.527	0.065	−0.193	0.200	0.428	0.353	0.529
M8	789	0.287	0.091	0.419	0.075	−0.227	0.283	0.409	0.294	0.504	0.062	−0.151	0.178	0.368	0.285	0.489
M8	79	0.286	0.089	0.419	0.066	−0.224	0.275	0.374	0.247	0.475	0.070	−0.133	0.172	0.217	0.129	0.322
M8	89	0.265	0.090	0.348	0.282	0.156	0.442	0.422	0.346	0.491	−0.095	−0.283	0.038	0.451	0.391	0.564
M9	1	0.190	0.087	0.295	0.284	0.173	0.351	0.317	0.182	0.456	0.187	0.047	0.348	0.530	0.457	0.628
M9	2	0.190	0.087	0.295	0.224	0.119	0.379	0.369	0.270	0.528	0.200	0.085	0.327	0.605	0.500	0.669
M9	3	0.271	0.176	0.416	0.395	0.279	0.562	0.471	0.370	0.661	0.374	0.219	0.475	0.618	0.493	0.705
M9	4	0.320	0.209	0.434	0.432	0.327	0.611	0.461	0.350	0.601	0.379	0.267	0.496	0.632	0.520	0.708
M9	5	0.367	0.236	0.461	0.427	0.274	0.634	0.496	0.325	0.627	0.346	0.235	0.498	0.625	0.517	0.698
M9	6	0.366	0.230	0.462	0.514	0.429	0.626	0.517	0.421	0.622	0.480	0.387	0.536	0.612	0.451	0.702
M9	7	0.433	0.277	0.539	0.691	0.616	0.776	0.537	0.452	0.599	0.453	0.335	0.591	0.548	0.467	0.607
M9	8	0.365	0.241	0.438	0.446	0.350	0.627	0.477	0.376	0.604	0.379	0.259	0.507	0.596	0.490	0.691
M9	9	0.452	0.341	0.592	0.717	0.648	0.825	0.518	0.398	0.600	0.447	0.317	0.615	0.445	0.321	0.557
M9	6789	0.436	0.314	0.542	0.665	0.592	0.763	0.557	0.448	0.666	0.451	0.357	0.544	0.599	0.500	0.671
M9	789	0.422	0.321	0.522	0.683	0.612	0.785	0.558	0.468	0.658	0.447	0.316	0.579	0.599	0.502	0.678
M9	79	0.457	0.311	0.563	0.713	0.638	0.810	0.543	0.456	0.616	0.455	0.324	0.622	0.519	0.452	0.602
M9	89	0.425	0.327	0.521	0.690	0.623	0.799	0.541	0.446	0.657	0.430	0.325	0.544	0.599	0.498	0.701

## Appendix 2

See Table 4.

**Table 4:** Average Pearson correlation (APC), quantile 2.5% (LL) and quantile 97.5% (UL) for the testing set between the observed GY and the predicted GY using all the bands as predictor variables in each of the environments

Method	Time	Melgas			Drought			Irrigated			Red.Irrigated			EarlyHeat		
		APC	LL	UL	APC	LL	UL	APC	LL	UL	APC	LL	UL	APC	LL	UL
M10	1	0.070	−0.073	0.187	0.205	0.139	0.259	0.132	−0.004	0.274	0.060	−0.088	0.181	0.488	0.320	0.565
M10	2	0.070	−0.073	0.187	0.183	0.065	0.325	0.291	0.124	0.449	0.172	0.084	0.295	0.493	0.399	0.570
M10	3	0.138	0.020	0.285	0.325	0.260	0.413	0.388	0.186	0.567	0.332	0.177	0.457	0.540	0.487	0.609
M10	4	0.242	0.099	0.339	0.397	0.321	0.500	0.378	0.289	0.527	0.294	0.152	0.441	0.530	0.453	0.564
M10	5	0.268	0.132	0.401	0.369	0.250	0.499	0.396	0.309	0.505	0.240	0.105	0.407	0.572	0.448	0.667
M10	6	0.194	−0.054	0.290	0.478	0.338	0.582	0.361	0.209	0.443	0.395	0.288	0.505	0.549	0.437	0.671
M10	7	0.326	0.175	0.451	0.687	0.548	0.789	0.454	0.368	0.546	0.396	0.316	0.465	0.542	0.427	0.640
M10	8	0.289	0.225	0.395	0.434	0.311	0.507	0.392	0.283	0.501	0.288	0.201	0.367	0.525	0.374	0.620
M10	9	0.368	0.204	0.567	0.682	0.583	0.758	0.397	0.202	0.500	0.364	0.248	0.472	0.431	0.333	0.502
M10	6789	0.402	0.298	0.485	0.719	0.652	0.783	0.489	0.412	0.563	0.409	0.339	0.539	0.573	0.461	0.646
M10	789	0.410	0.283	0.506	0.710	0.620	0.770	0.505	0.388	0.571	0.375	0.272	0.446	0.559	0.472	0.624
M10	79	0.347	0.189	0.466	0.694	0.620	0.747	0.451	0.352	0.522	0.386	0.296	0.490	0.512	0.422	0.593
M10	89	0.400	0.326	0.484	0.711	0.640	0.789	0.483	0.368	0.556	0.371	0.271	0.462	0.538	0.405	0.641
M11	1	0.139	0.005	0.304	0.325	0.236	0.376	0.180	0.037	0.292	0.193	0.077	0.320	0.466	0.384	0.575
M11	2	0.139	0.005	0.304	0.250	0.115	0.446	0.217	0.079	0.318	0.238	0.073	0.404	0.430	0.281	0.587
M11	3	0.160	−0.031	0.289	0.442	0.311	0.579	0.474	0.353	0.613	0.389	0.285	0.467	0.310	0.163	0.396
M11	4	0.331	0.230	0.435	0.449	0.325	0.631	0.464	0.363	0.588	0.383	0.256	0.485	0.304	0.096	0.409
M11	5	0.287	0.102	0.419	0.450	0.320	0.646	0.458	0.276	0.541	0.359	0.283	0.486	0.251	0.031	0.412
M11	6	0.368	0.228	0.496	0.500	0.419	0.655	0.167	−0.011	0.261	0.482	0.406	0.541	0.264	0.088	0.393
M11	7	0.260	0.127	0.410	0.699	0.628	0.791	0.281	0.120	0.455	0.413	0.299	0.512	0.198	0.050	0.371
M11	8	0.370	0.276	0.465	0.441	0.336	0.662	0.336	0.203	0.444	0.411	0.320	0.492	0.225	0.021	0.337
M11	9	0.273	0.191	0.377	0.745	0.684	0.834	0.264	0.120	0.399	0.463	0.325	0.549	0.204	0.075	0.337
M11	6789	0.421	0.293	0.490	0.714	0.661	0.799	0.275	0.140	0.418	0.457	0.391	0.540	0.276	0.132	0.439
M11	789	0.388	0.286	0.448	0.723	0.668	0.804	0.320	0.201	0.467	0.458	0.375	0.535	0.274	0.193	0.429
M11	79	0.272	0.173	0.368	0.729	0.664	0.810	0.267	0.099	0.417	0.440	0.332	0.534	0.208	0.046	0.343
M11	89	0.432	0.331	0.502	0.687	0.635	0.781	0.364	0.227	0.494	0.444	0.364	0.544	0.360	0.229	0.490
M12	1	0.136	−0.023	0.266	0.316	0.242	0.366	0.167	0.025	0.298	0.204	0.081	0.331	0.460	0.372	0.573
M12	2	0.136	−0.023	0.266	0.247	0.111	0.478	0.219	0.126	0.307	0.246	0.073	0.393	0.426	0.279	0.584
M12	3	0.168	−0.057	0.317	0.446	0.316	0.594	0.476	0.354	0.618	0.390	0.282	0.476	0.326	0.188	0.429
M12	4	0.323	0.211	0.432	0.449	0.317	0.625	0.459	0.362	0.583	0.382	0.269	0.481	0.316	0.132	0.432
M12	5	0.285	0.109	0.426	0.448	0.301	0.640	0.454	0.274	0.540	0.354	0.284	0.485	0.294	0.078	0.452
M12	6	0.362	0.228	0.475	0.500	0.418	0.658	0.164	−0.003	0.304	0.476	0.400	0.540	0.298	0.188	0.400
M12	7	0.260	0.143	0.393	0.709	0.630	0.807	0.291	0.135	0.475	0.410	0.299	0.520	0.202	0.046	0.390
M12	8	0.366	0.243	0.476	0.455	0.334	0.660	0.332	0.176	0.439	0.404	0.323	0.485	0.244	0.100	0.365
M12	9	0.254	0.176	0.374	0.746	0.689	0.831	0.257	0.085	0.396	0.463	0.331	0.567	0.346	0.176	0.441
M12	6789	0.421	0.298	0.515	0.716	0.661	0.792	0.277	0.142	0.462	0.452	0.398	0.545	0.327	0.221	0.403
M12	789	0.382	0.278	0.451	0.723	0.667	0.799	0.330	0.223	0.511	0.457	0.362	0.545	0.355	0.239	0.459
M12	79	0.261	0.160	0.381	0.743	0.679	0.820	0.263	0.108	0.409	0.452	0.327	0.572	0.330	0.144	0.483
M12	89	0.430	0.331	0.499	0.720	0.655	0.796	0.353	0.224	0.500	0.456	0.343	0.552	0.375	0.263	0.521
M13	1	0.092	−0.040	0.234	0.312	0.230	0.358	0.137	0.005	0.282	0.173	0.034	0.311	0.467	0.368	0.572
M13	2	0.092	−0.040	0.234	0.229	0.078	0.457	0.206	0.089	0.298	0.253	0.136	0.363	0.415	0.263	0.558
M13	3	0.137	−0.084	0.300	0.440	0.323	0.590	0.472	0.355	0.626	0.396	0.253	0.487	0.313	0.195	0.404
M13	4	0.325	0.243	0.425	0.436	0.313	0.612	0.450	0.349	0.560	0.384	0.265	0.474	0.301	0.106	0.419
M13	5	0.273	0.111	0.394	0.443	0.302	0.601	0.446	0.274	0.545	0.344	0.275	0.478	0.288	0.077	0.496
M13	6	0.347	0.196	0.474	0.500	0.417	0.642	0.153	−0.015	0.322	0.466	0.383	0.541	0.291	0.138	0.372
M13	7	0.253	0.163	0.322	0.724	0.634	0.809	0.284	0.144	0.455	0.462	0.335	0.574	0.327	0.169	0.488
M13	8	0.361	0.230	0.476	0.452	0.329	0.639	0.312	0.174	0.420	0.402	0.340	0.480	0.231	0.099	0.368
M13	9	0.238	0.125	0.349	0.741	0.684	0.828	0.243	0.053	0.403	0.455	0.309	0.556	0.332	0.148	0.438
M13	6789	0.420	0.254	0.516	0.715	0.661	0.787	0.257	0.110	0.454	0.447	0.392	0.545	0.321	0.233	0.422
M13	789	0.381	0.235	0.479	0.720	0.665	0.797	0.307	0.192	0.490	0.454	0.370	0.545	0.348	0.240	0.446
M13	79	0.248	0.157	0.368	0.740	0.680	0.821	0.248	0.070	0.406	0.463	0.344	0.593	0.346	0.172	0.494
M13	89	0.425	0.314	0.486	0.728	0.662	0.811	0.338	0.204	0.494	0.455	0.343	0.557	0.366	0.250	0.491
M14	1	0.116	−0.015	0.286	0.291	0.232	0.349	0.126	0.006	0.307	0.145	0.004	0.287	0.489	0.322	0.599
M14	2	0.116	−0.015	0.286	0.222	0.091	0.420	0.200	0.081	0.337	0.267	0.166	0.429	0.399	0.251	0.531
M14	3	0.131	−0.142	0.332	0.430	0.332	0.565	0.466	0.339	0.606	0.394	0.257	0.502	0.297	0.156	0.404
M14	4	0.316	0.233	0.434	0.438	0.329	0.599	0.449	0.356	0.530	0.380	0.256	0.476	0.277	0.086	0.393
M14	5	0.261	0.065	0.378	0.444	0.280	0.608	0.441	0.291	0.525	0.326	0.255	0.452	0.292	0.076	0.503
M14	6	0.340	0.184	0.428	0.502	0.424	0.631	0.139	0.007	0.304	0.457	0.378	0.526	0.314	0.220	0.407
M14	7	0.247	0.135	0.340	0.727	0.641	0.809	0.276	0.172	0.478	0.456	0.331	0.565	0.348	0.213	0.511
M14	8	0.373	0.292	0.466	0.449	0.308	0.641	0.301	0.146	0.420	0.399	0.328	0.480	0.267	0.126	0.420
M14	9	0.234	0.109	0.400	0.740	0.680	0.831	0.227	0.030	0.378	0.455	0.328	0.541	0.363	0.201	0.457
M14	6789	0.420	0.251	0.493	0.722	0.668	0.789	0.240	0.102	0.435	0.439	0.387	0.501	0.348	0.249	0.428
M14	789	0.411	0.303	0.476	0.720	0.664	0.787	0.287	0.177	0.453	0.442	0.338	0.498	0.349	0.263	0.441
M14	79	0.262	0.145	0.403	0.737	0.676	0.806	0.221	0.040	0.385	0.465	0.369	0.555	0.349	0.198	0.481
M14	89	0.448	0.332	0.520	0.749	0.683	0.818	0.326	0.174	0.497	0.453	0.345	0.538	0.365	0.238	0.488
M15	1	0.119	0.010	0.288	0.273	0.194	0.337	0.125	−0.017	0.252	0.139	−0.038	0.281	0.484	0.331	0.593
M15	2	0.119	0.010	0.288	0.217	0.103	0.408	0.194	0.086	0.309	0.250	0.137	0.401	0.408	0.264	0.536
M15	3	0.131	−0.108	0.334	0.425	0.320	0.563	0.461	0.326	0.582	0.392	0.268	0.472	0.299	0.156	0.428
M15	4	0.327	0.249	0.409	0.433	0.315	0.577	0.436	0.347	0.528	0.378	0.237	0.470	0.276	0.037	0.410
M15	5	0.258	0.072	0.367	0.437	0.289	0.581	0.438	0.293	0.518	0.317	0.236	0.428	0.304	0.079	0.562
M15	6	0.336	0.171	0.447	0.508	0.416	0.626	0.146	0.033	0.254	0.454	0.340	0.534	0.313	0.221	0.420
M15	7	0.240	0.127	0.327	0.731	0.643	0.821	0.254	0.134	0.454	0.458	0.341	0.580	0.369	0.228	0.496
M15	8	0.377	0.298	0.467	0.452	0.297	0.635	0.283	0.132	0.395	0.387	0.317	0.473	0.253	0.074	0.393
M15	9	0.213	0.073	0.360	0.737	0.676	0.826	0.218	0.047	0.347	0.460	0.322	0.545	0.358	0.191	0.463
M15	6789	0.414	0.271	0.493	0.744	0.682	0.811	0.225	0.106	0.407	0.434	0.380	0.495	0.351	0.275	0.478
M15	789	0.410	0.297	0.488	0.729	0.669	0.788	0.276	0.143	0.432	0.437	0.326	0.511	0.339	0.270	0.406
M15	79	0.269	0.155	0.373	0.739	0.676	0.811	0.204	0.023	0.379	0.464	0.350	0.563	0.368	0.240	0.475
M15	89	0.452	0.329	0.540	0.759	0.680	0.840	0.314	0.159	0.476	0.455	0.322	0.536	0.363	0.245	0.480
M16	1	0.106	−0.007	0.268	0.261	0.182	0.351	0.110	0.032	0.202	0.119	−0.067	0.270	0.492	0.353	0.582
M16	2	0.106	−0.007	0.268	0.211	0.091	0.397	0.183	0.057	0.311	0.253	0.145	0.374	0.401	0.255	0.556
M16	3	0.107	−0.144	0.313	0.428	0.344	0.567	0.451	0.323	0.583	0.402	0.251	0.479	0.315	0.173	0.409
M16	4	0.321	0.193	0.426	0.431	0.310	0.564	0.429	0.351	0.528	0.381	0.255	0.477	0.279	0.052	0.441
M16	5	0.249	0.067	0.358	0.443	0.325	0.573	0.427	0.285	0.503	0.307	0.205	0.422	0.290	0.083	0.550
M16	6	0.321	0.156	0.445	0.505	0.421	0.626	0.129	0.033	0.239	0.456	0.332	0.558	0.306	0.214	0.432
M16	7	0.228	0.145	0.321	0.730	0.639	0.823	0.239	0.120	0.437	0.453	0.330	0.565	0.355	0.217	0.486
M16	8	0.376	0.306	0.474	0.496	0.380	0.616	0.271	0.122	0.382	0.375	0.315	0.462	0.248	0.087	0.379
M16	9	0.219	0.061	0.331	0.735	0.675	0.827	0.211	0.008	0.333	0.449	0.303	0.535	0.371	0.205	0.488
M16	6789	0.425	0.297	0.490	0.751	0.687	0.812	0.214	0.107	0.384	0.462	0.410	0.555	0.369	0.280	0.527
M16	789	0.434	0.368	0.524	0.737	0.665	0.795	0.259	0.137	0.413	0.437	0.327	0.508	0.375	0.282	0.510
M16	79	0.256	0.160	0.331	0.739	0.678	0.806	0.181	−0.012	0.364	0.450	0.330	0.553	0.359	0.206	0.485
M16	89	0.460	0.353	0.555	0.757	0.678	0.835	0.303	0.173	0.449	0.444	0.312	0.519	0.356	0.249	0.476
M17	1	0.182	0.070	0.285	0.319	0.239	0.397	0.294	0.174	0.438	0.228	0.111	0.326	0.562	0.456	0.651
M17	2	0.182	0.070	0.285	0.298	0.205	0.466	0.396	0.296	0.580	0.302	0.153	0.467	0.605	0.530	0.671
M17	3	0.243	0.125	0.391	0.469	0.340	0.625	0.485	0.321	0.621	0.443	0.349	0.505	0.620	0.540	0.702
M17	4	0.344	0.267	0.436	0.475	0.361	0.626	0.473	0.331	0.619	0.399	0.290	0.461	0.609	0.553	0.699
M17	5	0.380	0.256	0.491	0.484	0.378	0.652	0.497	0.401	0.585	0.375	0.283	0.465	0.650	0.540	0.721
M17	6	0.357	0.177	0.476	0.573	0.515	0.683	0.486	0.387	0.604	0.485	0.410	0.539	0.630	0.499	0.696
M17	7	0.450	0.292	0.555	0.736	0.642	0.828	0.563	0.487	0.638	0.474	0.328	0.573	0.610	0.531	0.682
M17	8	0.382	0.287	0.443	0.540	0.429	0.653	0.479	0.367	0.611	0.414	0.319	0.498	0.589	0.527	0.666
M17	9	0.482	0.319	0.630	0.747	0.678	0.842	0.527	0.370	0.649	0.466	0.318	0.596	0.555	0.481	0.622
M17	6789	0.489	0.361	0.554	0.747	0.674	0.816	0.571	0.493	0.625	0.494	0.393	0.595	0.642	0.537	0.700
M17	789	0.501	0.364	0.589	0.745	0.683	0.817	0.573	0.475	0.643	0.482	0.389	0.581	0.647	0.531	0.735
M17	79	0.479	0.306	0.618	0.749	0.674	0.833	0.547	0.443	0.629	0.477	0.344	0.579	0.615	0.522	0.699
M17	89	0.491	0.356	0.575	0.763	0.698	0.842	0.542	0.435	0.634	0.474	0.365	0.565	0.629	0.530	0.720
M18	1	0.181	0.087	0.272	0.319	0.240	0.402	0.291	0.170	0.434	0.224	0.082	0.342	0.560	0.452	0.649
M18	2	0.181	0.087	0.272	0.306	0.210	0.466	0.398	0.300	0.562	0.295	0.154	0.478	0.602	0.528	0.676
M18	3	0.248	0.114	0.388	0.474	0.346	0.635	0.489	0.317	0.629	0.442	0.345	0.500	0.616	0.543	0.697
M18	4	0.344	0.260	0.429	0.486	0.360	0.633	0.479	0.364	0.597	0.409	0.279	0.476	0.612	0.548	0.700
M18	5	0.377	0.243	0.483	0.483	0.377	0.661	0.495	0.383	0.586	0.380	0.285	0.473	0.649	0.539	0.714
M18	6	0.360	0.188	0.472	0.570	0.501	0.674	0.494	0.364	0.610	0.485	0.414	0.540	0.631	0.506	0.698
M18	7	0.450	0.294	0.554	0.736	0.638	0.829	0.564	0.479	0.639	0.474	0.327	0.570	0.614	0.542	0.682
M18	8	0.382	0.296	0.449	0.531	0.404	0.655	0.483	0.361	0.615	0.422	0.334	0.510	0.593	0.532	0.668
M18	9	0.482	0.323	0.630	0.747	0.675	0.843	0.527	0.368	0.645	0.469	0.296	0.612	0.557	0.468	0.629
M18	6789	0.487	0.364	0.547	0.756	0.684	0.825	0.569	0.483	0.626	0.510	0.425	0.599	0.652	0.530	0.710
M18	789	0.503	0.376	0.591	0.750	0.676	0.821	0.572	0.464	0.648	0.503	0.390	0.597	0.652	0.532	0.735
M18	79	0.477	0.304	0.614	0.748	0.669	0.832	0.548	0.435	0.627	0.483	0.330	0.597	0.618	0.517	0.704
M18	89	0.497	0.375	0.589	0.764	0.702	0.840	0.544	0.429	0.642	0.491	0.355	0.591	0.630	0.527	0.725
M19	1	0.084	−0.073	0.184	0.230	0.174	0.296	0.156	0.021	0.280	0.085	−0.055	0.245	0.505	0.344	0.585
M19	2	0.084	−0.073	0.184	0.209	0.096	0.339	0.324	0.170	0.466	0.200	0.105	0.315	0.517	0.435	0.610
M19	3	0.148	0.041	0.304	0.395	0.326	0.518	0.400	0.186	0.587	0.363	0.227	0.470	0.559	0.505	0.630
M19	4	0.247	0.128	0.351	0.395	0.299	0.514	0.393	0.288	0.532	0.321	0.191	0.464	0.556	0.496	0.594
M19	5	0.277	0.168	0.381	0.372	0.263	0.500	0.413	0.303	0.536	0.240	0.122	0.375	0.600	0.483	0.681
M19	6	0.208	−0.058	0.340	0.475	0.341	0.570	0.390	0.250	0.489	0.417	0.284	0.527	0.585	0.459	0.680
M19	7	0.359	0.188	0.482	0.698	0.570	0.800	0.478	0.395	0.549	0.437	0.353	0.497	0.575	0.498	0.675
M19	8	0.299	0.248	0.378	0.448	0.280	0.534	0.391	0.305	0.486	0.307	0.227	0.385	0.564	0.449	0.627
M19	9	0.401	0.236	0.616	0.692	0.598	0.768	0.435	0.270	0.548	0.392	0.290	0.510	0.490	0.392	0.547
M19	6789	0.416	0.297	0.483	0.730	0.651	0.796	0.514	0.465	0.576	0.457	0.350	0.574	0.633	0.506	0.696
M19	789	0.441	0.344	0.521	0.723	0.627	0.789	0.519	0.429	0.597	0.435	0.342	0.518	0.623	0.514	0.679
M19	79	0.392	0.227	0.521	0.709	0.632	0.768	0.460	0.323	0.516	0.424	0.349	0.535	0.571	0.477	0.640
M19	89	0.434	0.388	0.494	0.720	0.649	0.795	0.495	0.389	0.583	0.402	0.294	0.529	0.581	0.479	0.649

## Appendix 3

See Figs. 9, 10, 11, 12, 13, 14, 15 and 16.

## Abbreviations

APC: average of the ten-fold cross-validation of the Pearson correlation
BLUP: best linear unbiased predictor
CIMMYT: Centro Internacional de Mejoramiento de Maíz y Trigo
CT: canopy temperature
DH: days to heading
GNDVI: green normalized difference vegetation index
GY: grain yield
H2: broad-sense heritability
HTPP: high-throughput phenotyping platforms
mND: modified normalized difference at 705 nm wavelength
NDVI: normalized difference vegetation index
NPQI: normalized pheophytinization index
OLS: ordinary least square
PC: principal components
PLS: partial least sqaure
PRI: photochemical reflectance index
RARSa: ratio analysis of reflectance spectra chlorophyll a
RARSb: ratio analysis of reflectance spectra chlorophyll b
RARSc: ratio analysis of reflectance spectra chlorophyll c
RNDVI: red normalized difference vegetation index
SRa: simple ratio
VI: vegetative index
